# Structure/function interrelationships and illness insight in patients with schizophrenia: a multimodal MRI data fusion study

**DOI:** 10.1007/s00406-023-01566-1

**Published:** 2023-02-20

**Authors:** Marie-Luise Otte, Mike M. Schmitgen, Nadine D. Wolf, Katharina M. Kubera, Vince D. Calhoun, Stefan Fritze, Lena S. Geiger, Heike Tost, Ulrich W. Seidl, Andreas Meyer-Lindenberg, Dusan Hirjak, Robert Christian Wolf

**Affiliations:** 1 Department of General Psychiatry, Center for Psychosocial Medicine, Heidelberg University, Vosstrasse 4, 69115 Heidelberg, Germany; 2 Department of Psychiatry and Psychotherapy, Central Institute of Mental Health, Medical Faculty Mannheim, Heidelberg University, Mannheim, Germany; 3 Department of Psychiatry and Psychotherapy, Research Group Systems Neuroscience in Psychiatry, Central Institute of Mental Health, Medical Faculty Mannheim, Heidelberg University, Mannheim, Germany; 4 Department of Psychiatry and Psychotherapy, SHG-Kliniken Saarbrücken, Saarbrücken, Germany; 5 Tri-Institutional Center for Translational Research in Neuroimaging and Data Science (TReNDS), Georgia State University, Georgia Institute of Technology and Emory University, Atlanta, GA USA

**Keywords:** Schizophrenia, MRI, Brain structure, Brain function, Parallel ICA, Illness insight

## Abstract

Illness insight in schizophrenia (SZ) has an important impact on treatment outcome, integration into society and can vary over the course of the disorder. To deal with and treat reduced or absent illness insight, we need to better understand its functional and structural correlates. Previous studies showed regionally abnormal brain volume in brain areas related to cognitive control and self-reference. However, little is known about associations between illness insight and structural and functional network strength in patients with SZ. This study employed a cross-sectional design to examine structural and functional differences between patients with SZ (*n* = 74) and healthy controls (*n* = 47) using structural and resting-state functional magnetic resonance imaging (MRI). Voxel-based morphometry was performed on structural data, and the amplitude of low frequency fluctuations (ALFF) was calculated for functional data. To investigate abnormal structure/function interrelationships and their association with illness insight, we used parallel independent component analysis (pICA). Significant group (SZ vs. HC) differences were detected in distinct structural and functional networks, predominantly comprising frontoparietal, temporal and cerebellar regions. Significant associations were found between illness insight and two distinct structural networks comprising frontoparietal (pre- and postcentral gyrus, inferior parietal lobule, thalamus, and precuneus) and posterior cortical regions (cuneus, precuneus, lingual, posterior cingulate, and middle occipital gyrus). Finally, we found a significant relationship between illness insight and functional network comprising temporal regions (superior temporal gyrus). This study suggests that aberrant structural and functional integrity of neural systems subserving cognitive control, memory and self-reference are tightly coupled to illness insight in SZ.

**Supplementary Information:**

The online version contains supplementary material available at 10.1007/s00406-023-01566-1.

## Introduction

Schizophrenia (SZ) is a relatively common psychiatric disorder with an estimated median lifetime prevalence of 0.48% [1–3]. SZ poses a large financial burden to society due to medical and socioeconomic costs (e.g., repeated hospitalizations, ambulatory medical care, higher prevalence of cardiovascular comorbidities, ambulatory nursery care, assisted living and unemployment) [4]. One of the core symptoms of SZ is lack of illness insight. Lack of illness insight is associated with higher relapse, increased hospitalization rate, longer duration of hospital stay, higher number of positive and negative symptoms as well as higher risk for suicide and aggressive acts against others [5–8]. From clinical perspective, illness insight can be divided into two subdomains: (1) clinical insight, describing the awareness of suffering from a disease, recognizing the symptoms related to the disease, associating them with the disease and accepting the necessity of a therapy, (2) cognitive insight, describing the ability to question own perceptions and concepts and if necessary to correct them [9].

In the last two decades, there has been an increase in neuroimaging studies seeking to better understand the neurobiological underpinnings of illness insight in SZ. Studies using magnetic resonance imaging (MRI) found regionally confined alterations in structural networks comprising frontal, parietal, temporal, basal ganglia, and cerebellar regions [9–14]. Further, abnormal brain activity at rest has been also related to illness insight in SZ. For instance, Gerretsen and co-workers found associations between symptom insight and self-referential processes ascribed to the so-called default mode network (DMN), together with associations between cognitive insight and the dorsal attention network [15]. In another study of resting-state activity, SZ patients’ samples were assigned to subgroups with good and poor insight, respectively. Special attention was paid to specific DMN subsystems, i.e., anterior and posterior DMN (aDMN, pDMN) [16], i.e., networks that are thought to subserve very distinct mnemonic and self-reflective processes [17]. Although qualitatively different aDMN and pDMN connectivity patterns were found between the patient groups, significant quantitative differences could not be confirmed. However, poor or strong illness insight in SZ patients cannot be explained by only one network alone. It is rather an interplay between different brain regions and networks, which can change their structure and function during the course of the disease. A previous meta-analysis of 21 neuroimaging studies on insight in psychosis conducted by Pijnenborg et al. [9] showed that clinical insight is not associated with abnormalities of isolated brain networks, but with rather diffuse global and frontal abnormalities responsible for cognitive and self-evaluative processes. The situation is different with cognitive insight, which is more dependent on individual brain regions crucial for retrieving and integrating self-related information [18].

It is important to note, that associations with illness insight have been reported for either brain structure or function, with both modalities predominantly assessing brain–behavior relationships on the regional level [9, 19]. Very little is known about associations between illness insight and structural or functional integrity at the neural system level. In addition, aberrant structure–function interrelationships and their effects on specific symptom expression (e.g., hallucinations or sensorimotor symptoms) have been increasingly reported in the past few years [20, 21], yet evidence relating such complex interactions to illness insight is lacking so far. To fill this gap, this study used a multivariate data analysis approach for multimodal data fusion, i.e., parallel independent component analysis (pICA) to identify maximally independent components of each imaging modality as well as the link between them [22, 23]. In particular, we chose amplitude of low frequency fluctuations (ALFF) given its more favorable retest reliability, following the report by Holiga and colleagues in 2018 [24]. While both measures (ALFF and fractional ALFF [fALFF]) exhibited moderate to highest–retest reliability within gray matter regions, reliability for ALFF tended to be higher than for fALFF. At least in part, this difference can be explained by the fact that fALFF is a proportional measure [25]. This finding suggests that ALFF is more reliable than fALFF in gray matter regions, and thus potentially more sensitive for discerning differences between individuals and groups [26]. Furthermore, we used the German version of the Osnabrück scale of adherence and identification of disease-related symptoms in schizophrenia (OSSTI) to measure illness insight [27, 28]. This instrument was chosen because it provides a separate analysis of two distinct dimensions of illness insight, i.e., identification of disease-related symptoms (OSSTI-I) and treatment adherence (OSSTI-A). Further, these two dimensions correspond well with the clinical insight model presented by David et al. [29, 30] and Amador et al. [31], which emphasizes (i) recognition of disease-related symptom as much as (ii) acceptance of treatment necessity [9].

Supporting a multi-dimensional neural model of illness insight influenced by aberrant networks linked to self-perception, self-awareness [32–34], and “anosognosia” [35–37], we predicted to find interactions between illness insight and both structural and functional network alterations in neural systems subserving cognitive control and self-referential processes.

## Methods

### Participants

Initially, 133 participants were included in this study. Twelve participants (2 HC, 10 SZ) were excluded during MRI data quality assurance: (a) 4 SZ and 1 HC due to low data quality of the structural MRI data (Participants with 2 or more standard deviations in the Mahalanobis distance were excluded. Mahalanobis distance was used as a measure which combines weighted overall image quality and mean correlation, which represent image artifacts like noise and motion before preprocessing and homogeneity of the data after preprocessing, respectively [38, 39]) and (b) 5 SZ and 1 HC due to excessive head movements (> 3 mm) during resting-state functional magnetic resonance imaging (rs-fMRI)). One hundred and twenty-one participants were considered for further analyses, i.e., 74 patients with SZ and 47 HC.

Recruitment of the participants with SZ took place in the Department of Psychiatry and Psychotherapy at the Central Institute of Mental Health in Mannheim, Germany. This study was conducted as part of a larger project on patients with schizophrenia spectrum disorders (SSD) [40]. As an attempt to minimize clinical heterogeneity and to present this in comparison to other studies on illness insight in SSD patients, we included only patients with a diagnosis of paranoid SZ according to ICD-10 (F20.0). Right-handedness and age between 18 and 65 years were further inclusion criteria. Patients with a history of any substance dependency except for tobacco were excluded. The HCs were recruited via personal communication and community advertisements. In the HC group inclusion criteria were right-handedness and absence of personal or family history of any mental disorder. The study was approved by the local ethics committees (Medical Faculties Mannheim and Heidelberg at Heidelberg University, Germany). Written informed consent was obtained from all participants after a detailed explanation of the aims and procedures of the study.

### Clinical assessment

The Positive and Negative Syndrome Scale (PANSS) was used for detailed psychometric assessment of symptoms related to SZ [41]. Participants with SZ were on a stable medication regime for at least 2 weeks, the daily dose of antipsychotic medication was calculated in olanzapine equivalents (OLZe) [42].

The OSSTI was used to measure illness insight in patients [27, 28]. This instrument was developed as a self-rating scale [27, 28], with explicit reference to three pre-existing insight rating instruments, i.e., Scale to Assess Unawareness of Mental Disorder (SUMD) [31], Birchwood's Insight Scale (BIS) [43] and the Self-Appraisal of Illness Questionnaire (SAIQ) [44]. The final version of the OSSTI consists of ten items as 6-point Likert scale, which are divided into two subdomains (OSSTI-A: items: 1, 3, 5, 7, 9, 10, OSSTI-I: items 2, 4, 6, 8, scores of subdomains were used in this study). Higher OSSTI scores refer to higher illness insight. An example for an item of OSSTI-A is: “After discharge of the hospital/ after moving out of the assisted living I will still be in the need of medical (psychiatric) or therapeutic care”. (Item 1) An example for OSSTI-I is: “There exist early warning signs for my mental illness.” (Item 4). Three items (2, 9, 10) are conceptualized with reverse polarity. Waldorf verified the OSSTI in a study on 85 patients with SZ [27]: A Cronbach’s α for standardized items of 0.79 for the whole OSSTI and a mean item intercorrelation of 0.28 was detected. Additionally, a significant correlation with the PANSS item G12 (lack of judgment and insight) of *r* = 0.54 (*p* < 0.001) was found. Finally, cognitive functioning was examined via Trail-Making Test B (TMT-B), the Symbol Digit Substitution Test (SDST), and the category fluency (CF) with animals fruits and vegetables as categories [45, 46]. Global functioning was assessed via Global Assessment of Functioning (GAF) [47]. However, these data are available for patients only (see supplementary table 2 for details).

### MRI data acquisition

Structural data were acquired using T1-weighted three-dimensional (3D) magnetization-prepared rapid gradient-echo at the Central Institute of Mental Health, Mannheim, Germany on a 3.0 Tesla Magnetom Tim Trio MRI scanner (Siemens Medical Systems) with the following parameters: flip angle 7°, echo time (TE) = 3.93 ms; repetition time (TR) = 2530 ms; inversion time (TI) = 1100 ms; FOV = 256 mm; slice plane = axial; slice thickness: 1 mm; resolution = 1.0 × 1.0 × 1.0 mm; number of slices 176.

For rs-fMRI, 167 whole-brain echo planar imaging (EPI) volumes were recorded in an axial orientation with the following imaging parameters: repetition time = 1790 ms, echo time = 28 ms, field of view = 192 × 192 mm, flip angle = 76°, voxel size = 3 × 3 × 3 mm, 34 slices, slice thickness = 3 mm. For rs-fMRI, all participants were instructed to lay still and with eyes closed, trying not to think of anything specific and neither to fall asleep. Rs-fMRI was followed by immediate verbal contact. A post-scanning exit interview was performed to verify adherence to these instructions.

### MRI data analysis

#### Preprocessing

For *structural data* analyses, the Statistical Parametric Mapping analysis package (SPM12 version 7771; www.fil.ion.ucl.ac.uk/spm/software/spm12/; last access: 27/11/2020) and the computational anatomy toolbox (CAT12 version vcat12.7; dbm.neuro.uni-jena.de/cat/; last access: 27/11/2020) as an extension of SPM12 were used in MATLAB (Version R2019b). Preprocessing included (a) data segmentation and normalization to a template space and segmentation into gray matter, white matter and cerebrospinal fluid, (b) slice display for visual inspection of the images for the manual data quality check, (c) estimation of total intracranial volume (TIV), (d) data quality check, where participants with two or more standard deviations in the Mahalanobis distance were excluded, and (e) smoothing via SPM12: 8 mm Full Width at Half Maximum (FWHM) smoothing was applied.

Preprocessing of *resting state-data (rs-data)* was performed using the Data Processing Assistant for Resting-State fMRI Advanced Edition (DPARSFA) [48]. For preprocessing of the raw rs-data into mean amplitude of low-frequency fluctuations (mALFF), the following steps were applied in DPARSFA: (a) removing of the first 10 time points (b) slice timing (using the middle slice as reference slice), (c) head motion correction (excessive head motion of > 3 mm or > 3 degrees between two volumes was set as an exclusion criterion, (d) regressing out of the nuisance covariates (mean signals from white matter and cerebrospinal fluid) and movement-related effects using the Friston 24-parameter model [49] and (e) normalization by DARTEL in Montreal Neurological Institute (MNI) space to 3 × 3 × 3 mm^3^, (e) spatial smoothing with a 6-mm FWHM isotropic Gaussian kernel, and (f) band-pass filtering (0.01–0.1 Hz) to reduce low-frequency drift and high-frequency noise. We chose the mALFF as a parameter for the following statistical analyses representing the rs-fMRI data.

#### Structural and functional data fusion

For pICA, the Parallel ICA Toolbox (ParaICATv1.0b) in MATLAB R2019b was used (http://trendscenter.org/software/fit). Two features were extracted for the following analysis: structural MRI (sMRI) data as gray matter volume (GMV) and rs-fMRI data as mALFF. Eight independent components (ICs) were estimated for each feature using the minimum description length (MDL). Within the pICA analysis the AA-type was used, which measures the correlation between mixing coefficient of both features. ICASSO was run 20 times to ensure the consistency of the ICs [50]. For visualization of the ICs, the spatial IC-maps of the Parallel ICA Toolbox were put into MRIcroGL v.1.2.20211006 (https://www.nitrc.org/projects/mricrogl) using thresholds of standard deviations |*z*|> 3.5 and overlay of an MNI-template. The Talairach Daemon database (http://www.talairach.org/daemon.html) was used to extract anatomical labels and stereotaxic coordinates for a threshold of |*z*|> 3.5 from the spatial IC-maps of the significant ICs of the previous analysis.

### Statistical analysis

Following statistical analyses were conducted and displayed offline using the R software environment for statistical computing (version 4.0.3; https://www.r-project.org/; last access: 15/11/2020, [51]). The descriptive figures of the demographics (violin plots) and the correlation graphs were performed using the R package ggplot2 [52]. Between-group differences (HC vs. SZ) were calculated using loading coefficients of sMRI and rs-fMRI ICs. Mann–Whitney-*U* Test, *t* test or Welch test (depending on value distribution, as assessed by the Shapiro–Wilk Test) and the homogeneity of variances (Levene test). Following the pathway for partial correlation of the R package ggm [4], two-tailed Pearson’s correlations were used to explore the relationships between OSSTI scores (total, OSSTI-A and OSSTI-I) and loading coefficients of sMRI and rs-fMRI ICs which showed significant between-group differences corrected for multiple comparison (Bonferroni corrected *p* value of 0.003). Age, sex, total PANSS-score, TIV (only in OSSTI vs GMV) and mean framewise displacement (FD) Power [40] (only in OSSTI vs mALFF) were used as covariates. A nominal significance threshold, a *p* value < 0.05 was chosen, followed by correction for multiple comparisons using Bonferroni correction (Bonferroni corrected *p* value of 0.001).

For completeness, we also performed correlation analyses using Spearman’s and Pearson’s correlation to examine the relationship between OSSTI-A, OSSTI-I and PANSS negative score as well as cognitive and global functioning in terms of TMT-B, SDST, CF and GAF scores.

## Results

### Demographics and clinical scores

Demographic and clinical details are summarized in Table 1. Violin plots with boxplots for mean and SD of OSSTI (OSSTI-total, OSSTI-I, OSSTI-A) and PANSS (total, positive, negative, and general) are shown in Fig. 1 of the supplementary material. There was a significant age difference between the two groups (*p* ≤ 0.001, Mann–Whitney-*U* Test) as well as a difference in education years (*p* < 0.001, Mann–Whitney-*U* Test). There was no gender difference between the groups.

**Table 1:** Demographic and clinical variables

	*SZ*	*HC*	*p* value
	*(n* = *74)*	*(n* = *47)*	
Age in years: mean (SD)	39.1 (10.9)	32.8 (10.8)	** < 0.001 + **
Range (lowest, highest value)	19, 65	19, 59	
Gender: male (%)	37 (50.0%)	20 (42.6%)	0.540^a^
Education years: mean (SD)	13.0 (2.4)	14.5 (1.6)	** < 0.001 + **
Range (lowest, highest value)	9, 19	9, 16	
Duration of illness in years: mean (SD)	10.1 (10.4)		
OLZe in mg: mean (SD)	17.6 (9.7)		
OSSTI-I: mean (SD)	12.7 (4.5)		
OSSTI-A: mean (SD)	19.9 (5.9)		
PANSS negative: mean (SD)	15.5 (7.1)		
PANSS positive: mean (SD)	15.6 (6.9)		
PANSS general: mean (SD)	33.4 (9.0)		
PANSS total: mean (SD)	64.4 (18.7)		

Significant *p* values (*p* < 0.05) are highlighted in bold font

Values presented as mean (standard deviation (SD)). Statistic refers to comparison between SZ and HC

*OLZe* Olanzapine equivalents, *OSSTI* Osnabrück scale of adherence and identification of disease-related symptoms in schizophrenia, *PANSS* Positive and Negative Syndrome Scale, *SD* standard deviation

^a^Chi-square-Test, + Mann–Whitney-*U* Test

**Fig. 1:**
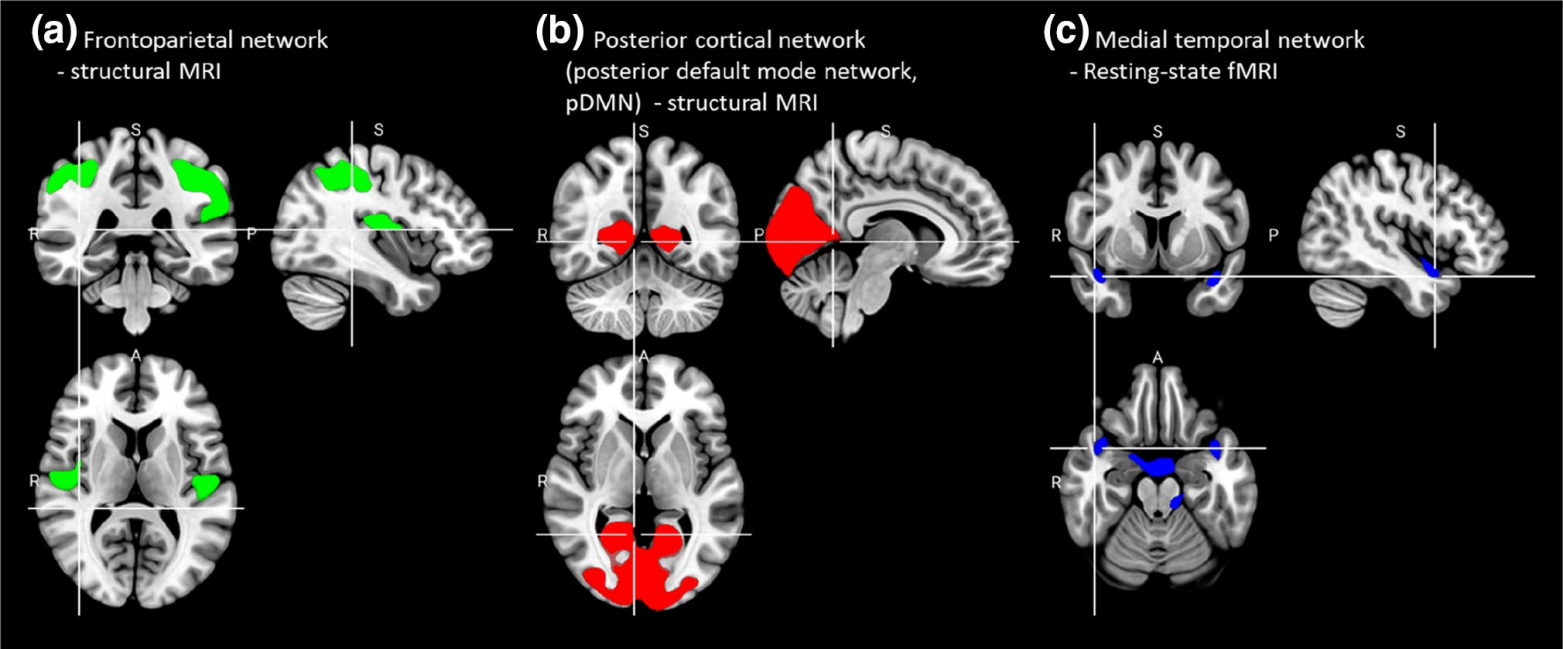
Spatial IC maps of Independent Components (ICs) showing a significant association with illness insight. Maps were overlayed on the MNI template (in sagittal, coronal, and axial slices) using thresholds of standard deviations |*z*|> 3.5

### Group differences

From a total of 8 structural and 8 functional components, we identified 12 networks that differed significantly between SZ patients and HC (*p* < 0.003, Bonferroni corr.), i.e., seven GMV. For details, see supplementary table 1.

### Associations between illness insight and independent components

**Fig. 2:**
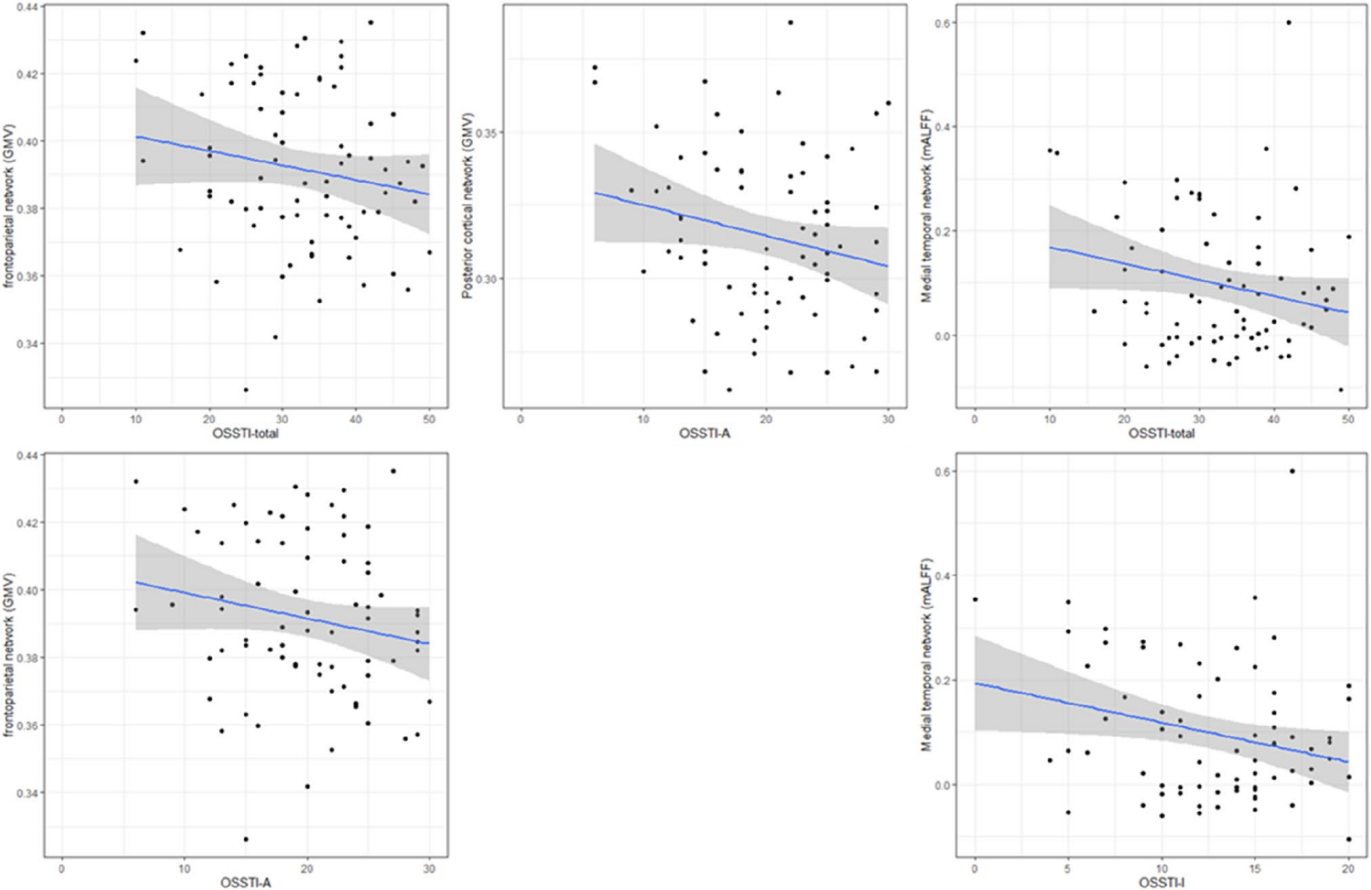
Correlation graphs of the significant correlations between the Independent Components (ICs) and OSSTI scores (OSSTI-total, OSSTI-I or OSSTI-A)

First, we identified a significant negative correlation between the structural frontoparietal network predominantly comprising the postcentral gyrus, inferior parietal lobule (IPL), precentral gyrus (M1), thalamus, precuneus, middle temporal gyrus (MTG), inferior frontal gyrus (IFG), superior temporal gyrus (STG), insula and middle frontal gyrus (MFG) and OSSTI total (OSSTI-total) (– 0.28, *p* = 0.021) and OSSTI-A (– 0.26, *p* = 0.028) scores (Figs. 1 and 2, Table 2). Second, we identified a significant negative correlation between structural posterior cortical midline structures comprising the cuneus, precuneus, lingual gyrus, posterior cingulate, and middle occipital gyrus and OSSTI-A scores (– 0.26, *p* = 0.030) (Figs. 1 and 2, Table 2). Third, the functional medial temporal network predominantly comprising the STG was significantly negatively associated with OSSTI-total (– 0.26, *p* = 0.027) and OSSTI-I (– 0.26, *p* = 0.027) scores (Figs. 1 and 2, Table 2). Finally, none of the *p* values remain significant after the Bonferroni correction (*p* < 0.001).

**Table 2:** Spatial distribution patterns of independent components showing differences between HC and SZ, as well as significant associations with illness insight in SZ

Feature	Brain region	Brodmann area	L	R	Volume (cc)
			*Z*-score	*Z*-score	
Frontoparietal	Postcentral Gyrus	1, 2, 3, 5, 7, 40, 43	11.5 (– 46, – 26, 37)	10.9 (56, – 15, 28)	6.3/8.6
network (GMV)	Inferior Parietal Lobule	2, 40	11.1 (– 43, – 27, 40)	10.0 (33, – 47, 45)	8.6/6.9
	Precentral Gyrus	4, 6, 9, 13, 43, 44	7.6 (– 50, – 19, 34)	9.6 (52, – 17, 35)	0.8/4.3
	Thalamus	-	7.3 (– 3, – 11, 9)	7.9 (3, – 11, 9)	2.4/1.9
	Precuneus	7, 19	6.5 (– 30, – 47, 48)	7.6 (30, – 47, 48)	0.8/2.0
	Middle Temporal Gyrus	19, 20, 21, 39	7.2 (– 40, – 62, 21)	-	2.0/-
	Superior Parietal Lobule	7	6.9 (– 27, – 50, 43)	7.0 (33, – 50, 48)	0.8/1.2
	Inferior Frontal Gyrus	9, 47	6.9 (– 39, 10, 27)	-	1.8/-
	Superior Temporal Gyrus	13, 22, 39, 41, 42	6.7 (– 43, – 59, 20)	4.1 (43, – 54, 21)	2.1/0.5
	Insula	13	6.5 (– 48, – 25, 15)	6.7 (48, – 18, 15)	2.0/2.1
	Middle Frontal Gyrus	8, 9, 46	6.5 (– 39, 14, 26)	5.3 (40, 6, 38)	1.5/0.9
	Transverse Temporal Gyrus	41, 42	5.9 (– 49, – 20, 12)	5.9 (53, – 14, 12)	0.6/0.6
	Supramarginal Gyrus	40	4.7 (– 55, – 38, 32)	5.1 (56, – 41, 31)	0.3/1.0
	Superior Frontal Gyrus	6, 8	4.2 (– 15, 24, 53)	4.9 (24, 32, 33)	0.5/0.6
	Paracentral Lobule	5	-	4.5 (4, – 45, 58)	-/0.3
Posterior cortical	Cuneus	7, 17, 18, 19, 23, 30	10.4 (– 4, – 93, 5)	10.2 (4, – 74, 15)	13.0/12.7
network	Lingual Gyrus	17, 18, 19	9.9 (– 1, – 87, 2)	9.9 (0, – 88, – 3)	8.2/7.5
(GMV)	Posterior Cingulate	23, 29, 30, 31	9.4 (– 1, – 68, 14)	9.9 (24, – 61, 10)	3.5/3.6
	Parahippocampal Gyrus	30	5.9 (– 18, – 49, 4)	8.0 (21, – 49, 5)	0.3/0.4
	Middle Occipital Gyrus	18, 19	7.6 (– 7, – 95, 14)	7.9 (33, – 81, 8)	2.9/4.4
	Precuneus	7, 19, 23, 31	7.9 (– 1, – 67, 20)	7.9 (4, – 67, 20)	4.4/5.1
	Inferior Occipital Gyrus	17, 18, 19	6.9 (– 12, – 92, – 8)	5.0 (27, – 89, – 7)	0.4/1.0
	Middle Temporal Gyrus	21, 37	5.2 (– 56, – 45, – 5)	4.4 (40, – 74, 12)	0.6/0.3
	Fusiform Gyrus	19, 20	4.4 (– 43, – 31, – 16)	-	0.3/-
Medial temporal	Superior Temporal Gyrus	38	5.6 (– 39, 8, – 21)	5.6 (39, 11, – 21)	0.8/1.0
network (mALFF)	Culmen	-	5.5 (– 12, – 33, – 16)	-	0.3/-
	Uncus	34	-	4.7 (15, – 1, – 20)	-/0.2
	Parahippocampal Gyrus	34, 35	-	4.6 (18, – 30, – 9)	-/0.3
	Insula	13	-	3.8 (39, – 17, 4)	-/0.3

Clusters with z > 3.5 were linked to the Talairach Daemon database to provide anatomical labels and were converted into MNI space. The maximum z-value and MNI coordinates are provided for each hemisphere (L = left; R = right). The volume of voxels in each area is displayed in cubic centimeters (cc)

### Explorative correlation analyses

For completeness, further correlations between illness insight and further structural and functional networks were performed, i.e., for components that did not exhibit significant between-group differences. None of these analyses yielded significant findings. Finally, we did not find any significant associations between OSSTI scores and other clinical and neurocognitive variables (Spearman’s and Pearson’s correlation: all *p* values > 0.05), except of a correlation between GAF and OSSTI-A (Spearman’s correlation 0.27, *p* = 0.02).

## Discussion

The aim of this multimodal study was to examine the relationship between illness insight and brain structure and resting-state neural activity in SZ patients using pICA. Two main findings emerged: First, we found a significant correlation between illness insight and structural alterations (GMV) of a network comprising frontoparietal and posterior cortical regions. Second, we found a significant relationship between illness insight and medial temporal network activity at rest (mALFF).

First, the frontoparietal network plays an important role in goal-driven behavior, making it possible to react in a fast, accurate and flexible way [53]. The frontoparietal network has often been described to be impaired in SZ patients and hence, there is considerable evidence that frontoparietal dysfunction in SZ patients is also associated with negative symptoms and cognitive impairment [18, 54, 55]. Interestingly, previous sMRI studies using voxel-based morphometry (VBM) and cortical thickness found an association between the right dorsolateral prefrontal cortex (DLPFC), other prefrontal regions and illness insight in SZ [19, 56, 57]. This said, structural alterations of the frontoparietal network in SZ may not only lead to negative symptoms and cognitive deficits, but to impaired illness insight as well.

Second, we found a significant association between functional alterations of the medial temporal network and OSSTI total and OSSTI-I scores. This finding is noteworthy, because the medial temporal network is a subsystem of the DMN and has been shown to play an important role in memory retrieval and prospection [58–60]. Alterations in this network could cause difficulties for SZ patients when they are overthinking their thoughts, perceptions, and actions, and try to put them into the context of their past, presence, and future experiences. This said, SZ patients have less abilities to put their own thoughts and perceptions into the environmental context and are therefore not able to identify them correctly as a part of their psychotic experience.

Third, with the posterior cortical midline structures we identified a significant relationship between functional alterations of the posterior DMN (pDMN) and varying illness insight. PDMN is the second subsystem of the DMN and is involved in self-representation, emotion, and salience detection [61]. This finding is also in line with the study of Liemburg et al. [16] who found a disconnection between the anterior and posterior DMN in SZ patients with poor illness insight. However, these results did not reach statistical significance [16]. Interestingly, although for obvious reasons it is difficult to directly compare structural with functional MRI studies, previous sMRI studies performing VBM and surface-based morphometry (SBM) analyses found structural alterations in different regions belonging to this network especially in the precuneus and poor illness insight [9]. Overall, the inability to remember previous episodes (due to disturbances of the medial temporal network) and to correctly assess current symptoms (due to disturbances of the pDMN) might lead to reduced illness insight and related consequences.

Interestingly, we found an association between treatment adherence (OSSTI-A) and structural changes as well as disease-related symptoms (OSSTI-I) and functional changes. Because no neuroimaging studies to date have investigated the neuronal correlates in such a differentiated way, we might only speculate about the pathomechanism underlying these aspects. On one side, psychopathological symptoms and the patient’s ability to classify his/her symptoms as part of the disease can often fluctuate along the disease course. This can then be possibly reflected in the aberrant function of different brain networks. On the other side, therapeutic adherence is a more stable/persistent feature in patients with partial or full remission and therefore more attributable to structural and spatially more delineated changes of particular brain networks. The review and meta-analysis by Pijnenborg et al. [9] was also able to show that different forms of insight (clinical and cognitive) have different neuronal correlates. Pijnenborg et al. [9] showed that clinical insight is related to spatially diffuse abnormalities across the brain; whereas, cognitive insight is mainly associated with ventrolateral prefrontal cortex and hippocampal areas. Finally, the absence of significant correlations between OSSTI scores and neurocognitive variables is noteworthy, since it supports a model where illness insight is conceived as a distinct symptom domain that doesn’t entirely overlap with the cognitive domain.

The strengths of this study consist of the large sample size and the use of pICA for the analysis of structural and functional MRI data. This method makes it possible to outline specific networks individually and to combine rs-fMRI and sMRI data analyses. However, this study also has limitations: (a) The cross-sectional study design is a limitation, because clinical disease course, psychopathology, and illness insight may fluctuate over time. This said, a cross-sectional study design is able to capture only one specific moment of illness insight. Illness insight at this particular time point can be modulated by situational effects that are affected by different psychopathological symptoms. To minimize situational effects potentially driven by overall symptom severity, we used the PANSS total score as a covariate. In future studies, situational effects may be better counteracted by more fine-grained methodological approaches, e.g., ecological momentary assessments (EMA). Such techniques may be better suited to detect fluctuations within longer time periods. In addition, in the context of this study functional neuroimaging markers are temporally less stable than structural parameters. Still, this study can provide initial indications of structural and functional network changes underlying different domains of illness insight in SZ. Our results are also consistent with our initial hypotheses and current literature that aspects of illness insight, which are of different temporal stability (recognition of psychopathological symptoms and acceptance of therapy), are associated with different neuroimaging parameters. (b) Since we compared SZ patients with HC, we may have identified SZ-associated brain changes rather than the true neuronal correlates of illness insight. Therefore, we strongly acknowledge MRI studies comparing SZ patients with and without illness insight to identify true neuronal correlates of illness insight. (c) The comparison with previous MRI studies on illness insight is difficult because of the usage of different scales for the measurement of illness insight and MRI analysis methods. Therefore, it is important to form national and international collaborations and to use standardized protocols when examining neuronal correlates underlying illness insight in SZ and other psychiatric disorders. (d) The identified association between the network strength and OSSTI scores did not survive Bonferroni correction for multiple testing. While these associations may appear neurobiologically plausible, and consistent with previous MRI studies on insight in SZ [62–64], such findings need to be interpreted with appropriate caution.

## Conclusion

Illness insight is associated with aberrant structural and functional networks responsible for self-reflection, memory, and memory retrieval as well as internal mentation. These findings may yield valuable translational potential, since targeting such network dysfunctions, e.g., via specific psychotherapeutic interventions or non-invasive neuromodulation may well lead to improved illness insight in SZ and subsequently to more favorable clinical and psychosocial outcomes [65].

### Supplementary Information

Below is the link to the electronic supplementary material.

Supplementary file1 (DOCX 15 KB)

## Data Availability

Data will be made available upon request to the extent permitted by the requirements of the European General Data Protection Regulation (GDPR).

## Abbreviations

BIS: Birchwood’s Insight Scale
CF: Category fluency
DLPFC: Dorsolateral prefrontal cortex
EPI: Echo planar imaging
GAF: Global Assessment of Functioning
ICs: Independent components
MFG: Middle frontal gyrus
MRI: Magnetic resonance imaging
sMRI: Structural MRI
rs-fMRI: Resting-state functional MRI
M1: Precentral gyrus
DK40: Desikan–Killiany atlas
DMN: Default mode network
aDMN: Anterior DMN
pDMN: Posterior DMN
FDR: False Discovery Rate
FOV: Field of view
FD: Framewise displacement
FDR: False discovery rate
FWHM: Full width at half maximum
HC: Healthy controls
IFG: Inferior frontal gyrus
IPL: Inferior parietal lobule
mALFF: Mean amplitude of low frequency fluctuations
MDL: Minimum description length
MNI: Montreal neurological institute
MTG: Middle temporal gyrus
OLZe: Olanzapine equivalents
OSSTI: Osnabrück scale of adherence and identification of disease-related symptoms by schizophrenia (originally in German: Osnabrücker Skala zu Therapieeinstellung und Identifikation psychischer Beschwerden bei Schizophrenie)
OSSTI-A: Adherence domain of OSSTI
OSSTI-I: Identification of disease-related symptoms domain of OSSTI
OSSTI-total: Total score of the OSSTI
PANSS: Positive and Negative Syndrome Scale
pICA: Parallel independent component analysis
ROI: Region of interest
rs: Resting state
SAIQ: Self-Appraisal of Illness Questionnaire
SBM: Surface-based morphometry
SD: Standard deviation
SDST: Symbol Digit Substitution Test
SPM12: Statistical Parametric Mapping
STG: Superior temporal gyrus
SUMD: Scale to Assess Unawareness of Mental Disorder
SZ: Patients with schizophrenia
TE: Echo time
TI: Inversion time
TIV: Total intracranial volume
TMT-B: Trail-Making Test B
TR: Repetition time
VBM: Voxel-based morphometry

## Code availability

Codes will be made available upon scientifically reasonable request.
