# Supplementary material for: Structure/function interrelationships and illness insight in patients with schizophrenia: a multimodal MRI data fusion study
Source: Eur Arch Psychiatry Clin Neurosci. 2023 Feb 20;273(8):1703–13. doi: 10.1007/s00406-023-01566-1 (PMC10713778; doi:10.1007/s00406-023-01566-1)
Supplement: Supplementary file 1 — Supplementary file1 (DOCX 15 KB) [file 406_2023_1566_MOESM1_ESM.docx]

**Supplementary table 1:**

Significant results (after bonferroni-correction p<0.003) in bold. There were conducted depending on if there was a normally distributions and homogenenity the Wilcoxon-test, the t-test or the Welch-t-test.

IC: Independent Component, #t-test, *Welch-test,+Mann–Whitney U test

| IC | p-value |
| --- | --- |
| mALFF comp1 | 0.964+ |
| mALFF comp2 | **<0.001#** |
| mALFF comp3 | **<0.001#** |
| mALFF comp4 | **<0.001*** |
| mALFF comp5 | **<0.001*** |
| mALFF comp6 | **<0.001+** |
| mALFF comp7 | **<0.001+** |
| mALFF comp8 | 0.2839+ |
| smwp comp1 | **0.001#** |
| smwp comp2 | **<0.001#** |
| smwp comp3 | **<0.001#** |
| smwp comp4 | **<0.001#** |
| smwp comp5 | **<0.001#** |
| smwp comp6 | 0.984# |
| smwp comp7 | **<0.001#** |
| smwp comp8 | **<0.001#** |

**Supplementary table 2:** Cognitive functioning and global assessment of functioning

|  | *SZ* |
| --- | --- |
|  | *(n=74)* |
| *TMT-B: mean (SD)* | *109.5 (63.14)* |
| *SDST: mean (SD)* | *53.76 (17.89)* |
| *CF total: mean (SD)* | *45 (14.72)* |
| *GAF: mean (SD)* | *70.95 (16.06)* |

Abbreviations: SZ: Schizophrenia patients, TMT-B: Trail Making Test B, SDST: Symbol Digit Substitution Test,

CF: Category Fluency, GAF: Global Assessment of Functioning, SD: standard deviation.
